# Within the Hospitals

**Published:** 1901-08-31

**Authors:** 


### WITHIN THE HOSPITALS.

### THE ROYAL SOUTH HANTS AND SOUTHAMPTON HOSPITAL.

IN "THE HOSPITAL" of July 23, 1892, page 285, and again in "THE HOSPITAL" of September 28, 1895, page 451, we drew special attention to the condition of this hospital, then known as the Royal South Hants Infirmary, and strongly urged the Governors to lose no time in erecting a new wing. The site was ample, and presented no difficulties whatever. We urged that care should be taken to secure that new wards, when erected, should exhibit all the recent developments in modern hospital construction that they might be a model to other institutions, and reflect honour not only upon the institution but upon Southampton itself. The Governors have acted upon our suggestion, and we thoroughly inspected the new wing on a recent occasion. We paid a surprise visit at a time when the ordinary working condition of the hospital could best be seen, and we are glad to be able to report that we found the new wards excellent and perfectly administered. They were erected from the plans of Messrs. Young and Hall, and must have proved very costly, though the report for 1900 publishes no building account or other information except that there is an outstanding liability of £8,000 on the building fund. Their costly character is due in large measure to the length and size of the corridors, a circumstance which has been utilised by the architects to provide under the best conditions food stores and separate rooms for storing patients' clothes on an excellent system, and for the linen required for the wards. The moral effect of a modern building upon the administration of a hospital is well exhibited at Southampton. In the ward sculleries we found that plate baskets had been provided, with electro-plated forks and everything which would enable the nursing staff to serve the patients' meals in the most appetising manner. Cupboards, drawers, lavatories and every part of the wards and their appurtenances were magnificently kept. The sisters and nurses whom we spoke to, several of whom had been trained at the Royal South Hants Hospital under Miss Mollett, were alert, intelligent, and whole-heartedly interested in their work. Indeed, anybody, whether interested in hospitals or not, who is suffering from ennui or depression would soon be cured if they would take the trouble to visit Southampton and spend a little time in the new wards there with the nursing staff and patients. Altogether we congratulate the people of Southampton and the managers of this hospital upon the public spirit they have exhibited in erecting the new wing—a building which should be visited by anybody who is interested in the extension of an old or the erection of a new hospital.

### A Few Points.

We observe that in this institution it is the custom to appoint three visitors of the hospital for each month in the year. Of the three visitors two are lay governors, and one is a member of the medical profession. In practice the system works well; it is the one now followed by the Prince of Wales's Fund, and we commend it to general adoption all over the country.

The operation theatre, instruments, and everything connected with it, which are in charge of a sister trained in the hospital, who is a Southerner born and bred, were admirably kept. There was really nothing to criticise or find fault with, and the maximum of cleanliness throughout the Royal South Hants Hospital—a cleanliness of everything everywhere—reflects the greatest honour on Miss Mollett, one of the very ablest and most efficient of matrons, upon the committee, and upon the medical and nursing staff of the institution. The contrast between the operation theatre at the Royal South Hants Hospital and that of another county infirmary which has recently been built, where they have filthy guttapercha receptacles contantly in use when operations are going on, was most striking. It reveals by demonstration that unless a hospital is efficiently administered by the heads of its nursing department, the newest and most perfect buildings will soon assume a neglected and unattractive air, no matter how much money may have been spent on them originally. Sister Operation at Southampton, with whom we discussed the point, gave it as her opinion that men are "such untidy creatures" it is not be supposed that they can keep an operation theatre, its instruments and appurtenances anything like as well as an intelligent and devoted member of the opposite sex.

### A Nurse's Home Required.

Having done so well in regard to the new wing, we hope that the people of Southampton will speedily take in hand the erection of a nurses' home, which is urgently called for. The committee are fully alive to the present need for such a home. The twenty-two nurses on the staff are housed at present in a block of buildings contiguous to, but separated from, the hospital, which formerly consisted of an old house and three cottages. These are in a very poor state of repair; they afford most inadequate accommodation, and when converted originally they were looked upon as a temporary makeshift, and are fast becoming quite unfit for occupation by the nursing staff. The town of Southampton has greatly prospered in recent years. The erection of the new wing is a practical testimony to the high intelligence of the inhabitants generally, and we hope that one or two of the leading citizens may see their way like those of other large industrial centres to come forward and defray the cost, say £5,000 to £7,000, of a new nurses' home. At any rate we hope that everyone resident in Southampton and the neighbourhood who takes an interest in its progress and prosperity will make a point of inspecting the new wing at the Royal South Hants Hospital, because such a visit will not only excite much pleasurable interest, but it must also tend to instruct the majority in matters of hygiene and domestic management which cannot fail to prove of value and use to them in the management of their own households.

